# Investing in global measles and rubella elimination is needed to avert deaths and advance health equity

**DOI:** 10.1016/S2214-109X(22)00388-6

**Published:** 2022-10

**Authors:** Pratima L Raghunathan, Walter Orenstein

**Affiliations:** Accelerated Disease Control Branch, Global Immunization Division, Center for Global Health, Centers for Disease Control and Prevention, Atlanta, GA 30329, USA; Department of Medicine, Emory University School of Medicine, Atlanta, GA, USA

Is it feasible to eradicate measles and rubella globally? In response to a request from the 70th World Health Assembly in 2017, a group of experts concluded in 2020 that global eradication was technically feasible with sustained high coverage of two doses of measles- and rubella-containing vaccine, but the more realistic course was to accelerate regional progress towards measles and rubella elimination.^1^ Advances have been hard-fought and occasionally reversed in the six WHO regions that have established measles elimination goals and the four regions with rubella elimination goals. Measles and rubella have been verified as eliminated in 81 and 93 countries, respectively, as of 2020.^2,3^

Given this fluid global context, Amy Winter and colleagues, in *The Lancet Global Health*,^4^ modelled the probability of achieving measles and rubella elimination between 2018 and 2100 in 93 countries that currently bear the greatest measles and rubella burden. Five independent modelling groups collaborated on this tour-de-force effort to project the annual probability of measles and rubella elimination, conservatively defined in this report as 5 infections per million population, in each country under two different scenarios. The “business as usual” scenario maintained 2017 immunisation coverage levels in each country, continued the current rhythm of periodic supplemental national immunisation campaigns, and stipulated that no additional countries would introduce measles or rubella vaccines. The intensified investment scenario posited that, for each country that had not yet achieved elimination or 95% coverage for both measles and rubella, the unvaccinated population would decline at an annual compound growth rate, such that a country with 20% unvaccinated children in one year would reduce these “zero-dose children” to 19·1% in the next year. To estimate annual numbers of measles and rubella infections, measles deaths, and cases of congenital rubella syndrome in each of the 93 countries, five different models were run through 200 stochastic simulations to the year 2100.

Despite the diversity and complexity of the modelling approaches, the consortium obtained broadly convergent results. First, the two national rubella transmission models yielded robust evidence that, with intensified investment, rubella elimination is highly probable at national and global levels. Business-asusual conditions generated a high burden of rubella infections and cases of congenital rubella syndrome; individual countries that had introduced rubella vaccine were likely to eliminate infections, but reintroductions from countries without rubella vaccine lengthened the time to elimination. Second, global measles elimination cannot be achieved under business-as-usual conditions: the two national measles transmission models predict an annual estimated median of 17 million or 20 million measles infections and a sobering 441 000 or 469 000 preventable measles deaths per year. Notably, the two different models indicate that intensified investment would significantly decrease annual measles infections to 900 000 or 2·1 million, and mortality to 3000 or 28 000 deaths per year. Moreover, with intensified investment, greater numbers of countries would eliminate measles on an earlier timeframe.

Unfortunately, these models indicate that, in some countries, measles elimination will not be possible even with intensified investments: countries with >95% routine measles and rubella vaccine coverage may still require supplemental immunisation activities due to population growth and clusters of unvaccinated or under-vaccinated persons. Measles elimination is an unstable condition that necessitates constant vigilance against importations from areas with low routine immunisation coverage. Thus, sustaining regional and national measles elimination requires attention to equitable access to immunisations. The Nigeria subnational measles transmission model demonstrates the importance of ensuring equitable delivery and uptake of vaccines, focused on reaching the zero-dose children who are most vulnerable to disease. The probability of achieving measles elimination declines markedly when vaccination is not prioritised to geographical areas with low coverage rates or when receipt of a first dose of measles vaccine is correlated with the second, suggesting that zero-dose children are being neglected.

Winter and colleagues’ models are useful because they advance the immunisation field in three critical ways. First, the work demonstrates that achieving rubella elimination is feasible, likely to be sustained, and requires intensified investment. Rubella virus has a substantially lower transmission rate than measles, and thus a lower herd immunity threshold, which facilitates rubella elimination.^5^ It has therefore been previously argued that eliminating rubella may be more feasible in the near-term than eliminating measles, and that anticipated rapid progress with rubella elimination could spur measles elimination forward.^6^ This work may convince the African and Eastern Mediterranean regions—both with high burdens of congenital rubella syndrome—of the necessity to establish rubella elimination goals and to accelerate the introduction of rubella-containing vaccine in those countries that have not yet done so.

Second, this work demonstrates the evident need for intensified investments to drive reductions in measles and rubella morbidity and mortality, in order to achieve the Immunization Agenda 2030 (IA2030) impact goal of averting 50 million deaths during 2021–30.^7^ The COVID-19 pandemic’s dramatic damage to routine immunisation has caused global measles vaccination coverage to regress to 81% in 2021, leaving 24·7 million completely unprotected zero-dose children and 14·7 million underimmunised children who have received one dose.^8^ Thus, the baseline assumptions of the intensified investment scenario are already belied by 2021 data. To close these yawning immunity gaps, national supplemental immunisation activities are likely to be needed in countries with low immunisation coverage.

Finally, this work explicitly shows that attention to equity is essential to advance measles and rubella elimination: within countries, tailored approaches at subnational levels, including improved microplanning, are crucial to identify children who missed measles and rubella vaccination, possibly due to COVID-19 pandemic disruptions. Globally, coordinated investments in countries with the greatest preventable mortality, largest numbers of susceptible individuals, or lowest immunisation rates may be warranted. This work can also galvanise innovation in improving measles and rubella vaccine delivery and demand: implementation of five-dose vials to expand access,^9^ monitoring efficacy of the measles microneedle array patch technology in current clinical trials,^10^ and collaborating with underimmunised communities to bolster demand for immunisation are all potential ways to accelerate reductions in unimmunised populations.

A focus on measles elimination can generate benefits beyond measles incidence reductions by markedly improving routine immunisation or supplemental immunisation activities used to deliver other vaccines besides measles.^11^ Investing in measles and rubella elimination will be a long and winding path but, in line with the IA2030 goal to avert 5 million deaths per year this decade, it is necessary to save lives and advance global health equity.
